# Draft Genome Sequences of Seven Thermophilic Spore-Forming Bacteria Isolated from Foods That Produce Highly Heat-Resistant Spores, Comprising *Geobacillus* spp., *Caldibacillus debilis*, and *Anoxybacillus flavithermus*

**DOI:** 10.1128/genomeA.00105-16

**Published:** 2016-05-05

**Authors:** Erwin M. Berendsen, Marjon H. J. Wells-Bennik, Antonina O. Krawczyk, Anne de Jong, Auke van Heel, Siger Holsappel, Robyn T. Eijlander, Oscar P. Kuipers

**Affiliations:** a Molecular Genetics, University of Groningen, Groningen, The Netherlands; b NIZO food research, Ede, The Netherlands; c Top Institute Food and Nutrition (TIFN), Wageningen, The Netherlands

## Abstract

Here, we report the draft genomes of five strains of *Geobacillus spp.*, one *Caldibacillus debilis* strain, and one draft genome of *Anoxybacillus flavithermus*, all thermophilic spore-forming Gram-positive bacteria.

## GENOME ANNOUNCEMENT

Thermophilic spore-forming bacteria typically grow at temperatures above 45°C and produce spores that are highly resistant to environmental insults, including high temperatures. Thermophilic spore-formers are encountered in a range of different environments (1) and their spores may be able to survive intense heat treatments, including commercial food sterilization processes used for canning and ultra-high temperature processes applied in the manufacturing of liquid foods. Spores that survive such heat treatments may subsequently germinate, and subsequent growth may result in food spoilage (2–4). Here, we sequenced the genomes of five strains of *Geobacillus* spp. (*G. toebii, G. stearothermophilus*, and *Geobacillus* sp.), one strain of *Anoxybacillus flavithermus*, and one strain of *Caldibacillus debilis*. The genome sequences of these strains can provide information on the thermophilic lifestyle of these strains and on the ability of the strains to form spores with high-level heat resistance (5).

The seven strains were grown overnight in 10 ml brain heart infusion broth (Difco) at 55°C. Cultures were 100-fold diluted, cultivated at 55°C, and harvested by centrifugation (5,000 relative centrifugal force) at the exponential growth phase. Total DNA was isolated as described previously (5). The isolated DNA was sheared to 500-bp fragments in the Covaris (KBioscience) ultrasone device; a next-generation sequencing library was prepared using the paired-end NEB NExtGen library preparation kit. The libraries were 101-bp paired-end sequenced on an Illumina HiSeq2000 by multiplexing 12 samples per flow cell. Draft genome sequences of all strains were assembled *de novo* using Velvet (6). The genomes were annotated using the RAST server (7). Scaffolds were mapped on the closest genome according to RAST, using CONTIGuator (8). Potential bacteriocin gene clusters were identified using BAGEL3 (9) in strains B4109, B4113, B4114, and B4119. Finally, InterProScan (10) was used to extend protein annotations.

### Nucleotide sequence accession numbers.

The genome sequences of the seven thermophilic spore-forming strains have been deposited as whole-genome shotgun projects at DDBJ/EMBL/GenBank under the accession numbers listed in Table 1.

**TABLE 1:** Genome features and GenBank accession numbers of the strains

Strain	Species	Source of isolation	BioProject no.	Accession no.
B4109	*Geobacillus stearothermophilus*	Pea soup	PRJNA270597	LQYV00000000
B4110	*Geobacillus toebii*	Pea soup	PRJNA270597	LQYW00000000
B4113	*Geobacillus* sp.	Mushroom soup	PRJNA270597	LQYX00000000
B4114	*Geobacillus stearothermophilus*	Buttermilk powder	PRJNA270597	LQYY00000000
B4119	*Geobacillus caldoxylosilyticus*	Dairy	PRJNA270597	LQYS00000000
B4135	*Caldibacillus debilis*	Dairy	PRJNA270597	LQYT00000000
B4168	*Anoxybacillus flavithermus*	Dairy	PRJNA270597	LQYU00000000
